# Genome Analyses and Genome-Centered Metatranscriptomics of *Methanothermobacter wolfeii* Strain SIV6, Isolated from a Thermophilic Production-Scale Biogas Fermenter

**DOI:** 10.3390/microorganisms8010013

**Published:** 2019-12-20

**Authors:** Julia Hassa, Daniel Wibberg, Irena Maus, Alfred Pühler, Andreas Schlüter

**Affiliations:** Center for Biotechnology (CeBiTec), Bielefeld University, Genome Research of Industrial Microorganisms, Universitätsstraße 27, 33615 Bielefeld, Germany; jhassa@cebitec.uni-bielefeld.de (J.H.); dwibberg@CeBiTec.Uni-Bielefeld.DE (D.W.); irena.maus@cebitec.uni-bielefeld.de (I.M.); puehler@cebitec.uni-bielefeld.de (A.P.)

**Keywords:** *Methanothermobacter wolfeii*, thermophilic biogas fermenter, genome mining, comparative analyses, CRISPR/*cas*, metabolic pathway reconstruction, metagenomics, fragment recruitment, metatranscriptomics

## Abstract

In the thermophilic biogas-producing microbial community, the genus *Methanothermobacter* was previously described to be frequently abundant. The aim of this study was to establish and analyze the genome sequence of the archaeal strain *Methanothermobacter wolfeii* SIV6 originating from a thermophilic industrial-scale biogas fermenter and compare it to related reference genomes. The circular chromosome has a size of 1,686,891 bases, featuring a GC content of 48.89%. Comparative analyses considering three completely sequenced *Methanothermobacter* strains revealed a core genome of 1494 coding sequences and 16 strain specific genes for *M. wolfeii* SIV6, which include glycosyltransferases and CRISPR/*cas* associated genes. Moreover, *M. wolfeii* SIV6 harbors all genes for the hydrogenotrophic methanogenesis pathway and genome-centered metatranscriptomics indicates the high metabolic activity of this strain, with 25.18% of all transcripts per million (TPM) belong to the hydrogenotrophic methanogenesis pathway and 18.02% of these TPM exclusively belonging to the *mcr* operon. This operon encodes the different subunits of the enzyme methyl-coenzyme M reductase (EC: 2.8.4.1), which catalyzes the final and rate-limiting step during methanogenesis. Finally, fragment recruitment of metagenomic reads from the thermophilic biogas fermenter on the SIV6 genome showed that the strain is abundant (1.2%) within the indigenous microbial community. Detailed analysis of the archaeal isolate *M. wolfeii* SIV6 indicates its role and function within the microbial community of the thermophilic biogas fermenter, towards a better understanding of the biogas production process and a microbial-based management of this complex process.

## 1. Introduction

Energy generation from renewable sources is an important part of the energy transition, taking climate protection into account. Anaerobic digestion (AD) processes of agricultural waste and renewable biomass with subsequent utilization of produced biogas for energy generation represents one of the opportunities in industrialization of agriculture and nowadays is in the focus of multiple investigations. AD of organic materials to biogas is a highly complex process and only the principles of involved degradation pathways are well-known so far [1,2]. A heterogeneous microbial community composed of members of the domains *Bacteria* and *Archaea* is responsible for anaerobic biomass decomposition [3,4] with the capacity to produce methane mainly by members of the phylum *Euryarchaeota* and few other phyla (e.g., *Bathyarchaeota*, *Verstraetearchaeota*) within the domain *Archaea* [5]. Process performance and composition of biogas microbiomes were described to be affected by the type of substrates and fermenter temperature in particular [4,6,7]. Most of the German full-scale biogas fermenters are operated under mesophilic (37–42 ${}^{\circ }C$) conditions; only few fermenters, i.e., 6%, perform the biomethanation under thermophilic (52–56 ${}^{\circ }C$) conditions [8]. In this context, studies addressing taxonomic analyses of the archaeal diversity within thermophilic biogas fermenters frequently described the occurrence of the genus *Methanothermobacter* [8,9,10,11], leading to the assumption that *Methanothermobacter* members contribute to a stable and well running thermophilic anaerobic digestion process. According to the ’list of prokaryotic names with standing in nomenclature’ (LPSN) [12], the genus *Methanothermobacter* currently comprises eight known species, namely *M. crinale* [13], *M. defluvii* [14], *M. marburgensis* [15], *M. thermautotrophicus* [16], *M. thermoflexus* [14], *M. thermophilus* [17], *M. tenebrarum* [18] and *M. wolfeii* [19], most of them originating from sewage sludge environments, gas-associated formation water or oil fields. All strains were described to be non-motile rods, Gram-positive (with the exception of the species *M. thermoflexus* being Gram-negative) and having an optimal growth temperature ranging from 55 to 70 ${}^{\circ }C$ [18]. All known *Methanothermobacter* species utilize carbon dioxide (CO${}_{2}$) and hydrogen (H${}_{2}$) as substrates for methanogenesis and hence perform the hydrogenotrophic pathway for methane production. Additionally, some species require acetate, yeast extract, cysteine or coenzyme M for growth [18]. To date, the genomes of three *Methanothermobacter* strains were published as complete chromosomes. These are *Methanothermobacter* sp. CaT2 [20], originating from gas-associated formation water in Japan, *M. thermautotrophicus*ΔH [21] and *M. marburgensis* str. Marburg [15], both isolated from sewage sludge. The prevalence of *Methanothermobacter* members in thermophilic AD communities raises the question of whether specific genome features may explain their competitiveness in AD environments. Thus, this study addresses the identification of genetic determinants potentially specifying competitiveness of *Methanothermobacter* species in the biogas fermenter habitat. For this purpose, the genome sequence of the strain *M. wolfeii* SIV6, isolated from a German thermophilic production-scale biogas fermenter [8] utilizing maize silage and pig manure for biomethanation, was established and analyzed in detail. Genome-centered metatranscriptome analyses provided insights into the transcriptional activity of *M. wolfeii* SIV6 genes under in situ conditions in the fermenter environment to assess the strain’s physiology and ecological adaptation. Moreover, compilation of the *M. wolfeii* SIV6 genome sequence enabled determination of its occurrence in biogas fermenter environments by applying metagenome fragment recruitments. It is expected that insights from this study can be exploited to optimize the final phase, the methanogenesis, of the anaerobic digestion process.

## 2. Materials and Methods

### 2.1. Isolation of the Strain M. wolfeii SIV6 from the Thermophilic Biogas Fermenter

*M. wolfeii* SIV6 was obtained from a thermophilic industrial-scale biogas fermenter (54 ${}^{\circ }C$) located in Viersen (Lat: 51.255499 N; Lon: 6.396524 E), North-Rhine-Westphalia (Germany). In this biogas fermenter, about 60% maize silage, 30% grass silage and 10% pig manure were used as substrates for biomethanation. Further details about the biogas fermenter parameters were described previously by Stolze et al. (2016) [4] and Maus et al. (2016) [8]. For the strain isolation, as well as metagenome and metatranscriptome sequencing, about 500 ml of fermentation sample was taken from the main fermenter. The isolation of *M. wolfeii* SIV6 from the fermenter sample was described before by Maus et al. (2016) [8] in the isolation strategy no. 11. Briefly, the strain was isolated via the deep agar shake method by using the DSMZ 287 medium supplemented with an amino acid solution incubated at 55 ${}^{\circ }C$. The cultivation techniques for targeting strict anaerobes described by Balch et al. (1979) [22] were used with a selective cultivating temperature, e.g., 65 ${}^{\circ }C$ and a combination of the antibiotics ampicillin and vancomycin.

### 2.2. Sequencing, Assembly and Annotation of the M. wolfeii SIV6 Genome

For genome sequencing of the isolate *M. wolfeii* SIV6, its genomic DNA was obtained applying the GeneMATRIX Stool DNA Purification Kit (Roboklon, Germany). A sequencing library (with an average paired-end distance of 760 bp) was constructed and sequenced on the Illumina MiSeq system applying the 2 × 300 bp paired-end protocol. The obtained reads were assembled by means of the GS *de novo* Assembler software (version 2.8, Roche), followed by genome finishing applying the CONSED software package [23] and an in silico gap closure approach as described previously by Wibberg et al. (2011) [24]. The annotation of the genome was accomplished within the GenDB 2.0 platform [25] and PROKKA 1.11 [26]. Additionally, the genome was screened for genomic island regions, pathogen-associated genes, virulence factors and antibiotic resistance genes with IslandViewer 4 [27] and CARD (Comprehensive Antibiotic Resistance Database [28]). Assignment of COG (Clusters of Orthologous Group) categories was done with WebMGA [29]. The genome sequence of *M. wolfeii* SIV6 is deposited in EBI database under the accession number LT608329. The type strain *Methanothermobacter wolfeii* is available from the Leibniz Institute `German Collection of Microorganisms and Cell Cultures’ (DSMZ, Braunschweig, Germany) under the accession number 2970.

### 2.3. Comparative Analyses of Methanothermobacter Genome Sequences

The genus *Methanothermobacter* currently consists of eight known species (Table 1) according to the ’list of prokaryotic names with standing in nomenclature’ [12], namely *M. crinale*, *M. defluvii*, *M. marburgensis*, *M. thermautotrophicus*, *M. thermoflexus*, *M. thermophilus* , *M. tenebrarum* and *M. wolfeii*.

The 16S rRNA gene sequences of these *Methanothermobacter* species, publicly available in the NCBI database, were used for the calculation of a 16S rRNA gene based maximum-likelihood phylogenetic tree with bootstrapping (1000 replications) applying MEGA X [30]. Only three *Methanothermobacter* strains, namely *Methanothermobacter* sp. CaT2 [20], *M. thermautotrophicus* ΔH [21] and *M. marburgensis* str. Marburg [15], were completely sequenced so far. These strains were used for comparative analyses within EDGAR [31], a platform for comparative analyses of prokaryotic genomes. These analyses include the calculation of the core genome, which are orthologous genes present in all or a subset of the compared strains, which are identified as reciprocal best blast hits and the identification of single genes (singletons) of the strain *M. wolfeii* SIV6, which are genes without a match, above the in EDGAR automatically calculated cutoff, with any of the reference genomes. Finally, average nucleotide identity (ANI) values [32] between the genome sequences of the reference strains and *M. wolfeii* SIV6 were calculated.

### 2.4. Metatranscriptome Mapping

To analyze the transcriptional activity of *M. wolfeii* SIV6 within the biogas fermenter, a mapping of metatranscriptome reads obtained from the corresponding biogas fermenter was accomplished. Total microbial RNA extraction and metatranscriptome sequencing was done as described in detail by Stolze et al. (2018) [33]. The RNA was isolated from the same fermenter sample as the *M. wolfeii* SIV6 strain. In brief, cDNA library preparation and metatranscriptome sequencing was done in two biological replicates on the Illumina HiSeq 2000 platform using the v3 chemistry (Illumina, USA) and following the 2 × 150 bp paired-end protocol at the DOE Joint Genome Institute (JGIWalnut Creek, CA, USA). The raw metatranscriptome sequencing data is available in the GOLD database [34] with biosample IDs Gb0054941 and Gb0054942. The metatranscriptome reads were quality trimmed with the tool trimmomatic v 0.35 [35] and aligned to the *M. wolfeii* SIV6 genome by means of Bowtie2 (default settings) [36]. Finally, transcripts per million (TPM), which were normalized by gene length and dataset size, were calculated for each gene within the *M. wolfeii* genome with ReadXplorer v2.2.3 [37]. Afterwards, a mapping of the genes with their TPM on the hydrogenotrophic methanogenesis pathway originating from the KEGG database was accomplished with KAAS [38].

### 2.5. Metagenome Fragment Recruitment

To calculate the abundance of *M. wolfeii* SIV6 within the corresponing thermophilic biogas fermenter, the metagenome reads of this biogas fermenter were mapped on the *M. wolfeii* genome. The total community DNA was obtained from the same sample of the biogas fermenter as strain SIV6. For total community DNA isolation, the CTAB-based chloroform-isoamyl alcohol DNA extraction method as described by Schlüter et al. (2008) [39] was used. Paired-end libraries were prepared by means of the Illumina TruSeq SBS v3-HS kit. Sequencing of the metagenome was performed in two biological replicates on the Illumina HiSeq 2000 sequencer following the 2 × 150 bp indexed high-output run protocol at the DOE Joint Genome Institute. The raw metagenome sequencing data is available in the GOLD database [34], with biosample IDs Gb0056840 and Gb0056841. Fragment recruitment was performed as described previously by Eikmeyer et al. (2013) [40]. Metagenomic reads were aligned to the *M. wolfeii* SIV6 genome by means of BLASTn. A minimum sequence identity of 55%, 75% and 97% with a minimum coverage of 90% were used as thresholds for the alignments. Finally, the fragment recruitment was visualized by plotting the identity of the alignment against the alignment position on the genome sequence of *M. wolfeii* SIV6.

## 3. Results and Discussion

### 3.1. General Genome Features of M. wolfeii Strain SIV6

**Figure 1:**
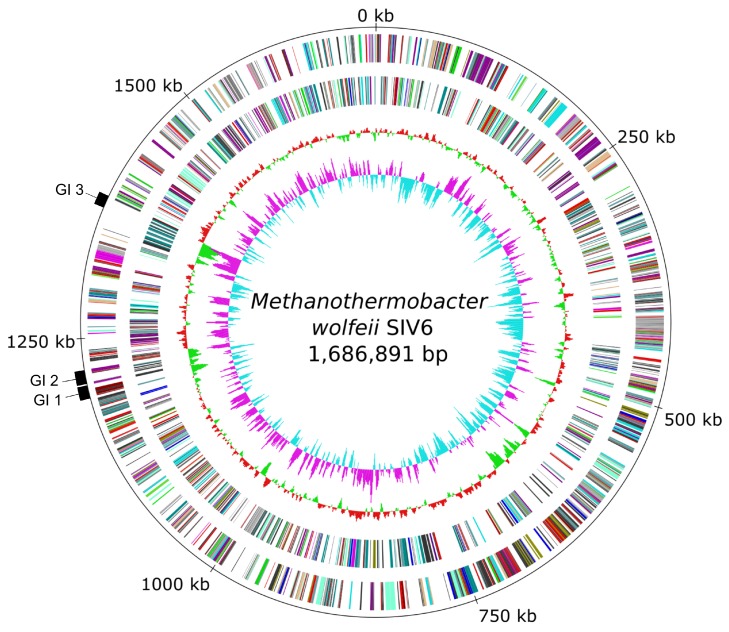
Circular genome plot and identified genomic islands (GIs) of the strain *M. wolfeii* SIV6. From the inner to the outer circle: Circle 1—GC skew; Circle 2—GC-content; Circle 3—predicted protein coding sequences transcribed anticlockwise colored according to the assigned COG (Clusters of Orthologous Group) categories; Circle 4—predicted protein coding sequences transcribed clockwise colored according to the assigned COG categories; Circle 5—genomic position in kb and identified genomic islands (GI 1–3).

Sequencing of the *M. wolfeii* SIV6 genome on the Illumina MiSeq platform resulted in 993,437 reads, accounting for 262,342,581 bases of total sequence information. The assembly resulted in 5 scaffolds comprising 87 contigs. Finally, the in silico finishing approach led to a closed circular *M. wolfeii* SIV6 chromosome with a size of 1,686,891 bases, featuring a GC content of 48.89% and a 150-fold genome coverage (Table 2, Figure 1). The gene prediction revealed 1659 protein coding sequences, 36 tRNA genes and two *rrn* operons.

In the genome of *M. wolfeii* SIV6, three genomic island regions (GI 1–3:, Figure 1) were identified with IslandViewer 4 [27]. Corresponding results are described in Section 3.3.3. Additionally, no pathogen-associated genes, virulence determinants, antibiotic resistance genes or phage-associated genes were identified within the genome with IslandViewer 4, CARD (Comprehensive Antibiotic Resistance Database) [28] or PHASTER [41]. The finished genome sequence of *M. wolfeii* SIV6 was deposited in the EBI database under the accession number LT608329.

### 3.2. Phylogenetic Classification as Deduced from Comparative Genome Analyses

**Figure 2:**
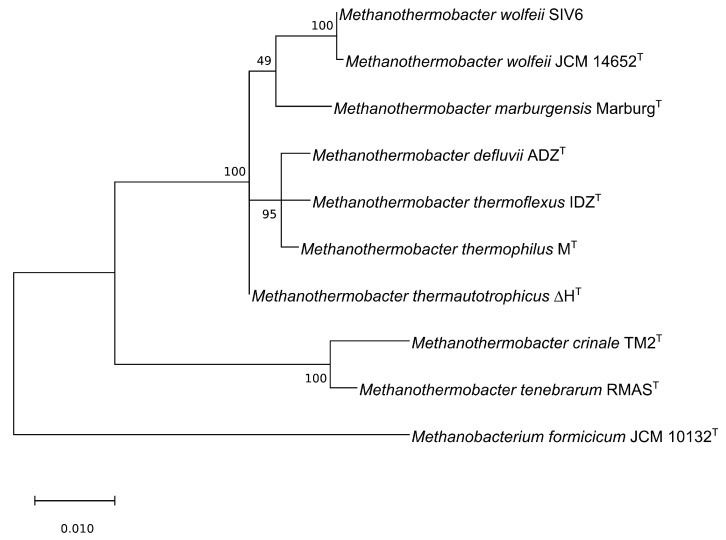
Maximum-likelihood phylogenetic tree based on the 16S rRNA gene sequences of all known *Methanothermobacter* type species according to the ‘list of prokaryotic names with standing in nomenclature’ (LPSN [12]) in comparison with *M. wolfeii* SIV6 and one outgroup, namely *Methanobacterium formicicum*, generated with MEGA X [30].

Currently, the genus *Methanothermobacter* comprises eight known type species according to LPSN [12] (Table 1). To determine the phylogeny of *M. wolfeii* SIV6 in relation to these *Methanothermobacter* species, a 16S rRNA gene based maximum-likelihood phylogenetic tree was calculated (Figure 2). The *M. wolfeii* SIV6 clusters together with the *M. wolfeii* type strain (Accession number: AB104858) [19]. The full-length 16S rRNA gene sequence comparison of strain SIV6 with the *M. wolfeii* type strain resulted in a 99% sequence identity.

**Figure 3:**
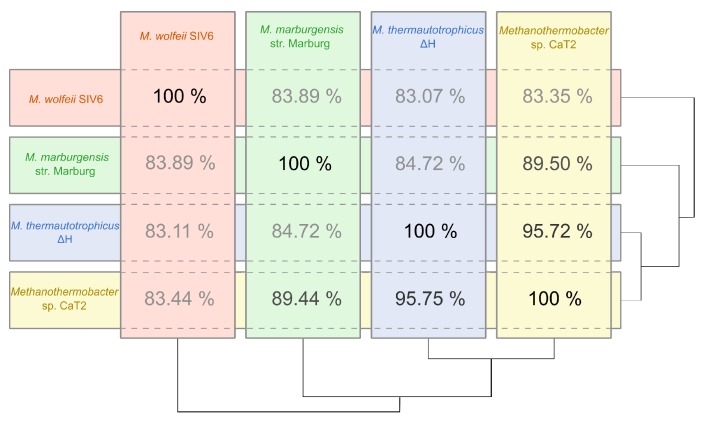
ANI (average nucleotide identity) diagram of *M. wolfeii* SIV6 and the reference strains *Methanothermobacter* sp. CaT2, *M. thermautotrophicus* ΔH and *M. marburgensis* str. Marburg based on calculations within the EDGAR [31] platform.

On the 16S rRNA gene level, the closest relatives of *M. wolfeii* SIV6 are *M. thermautotrophicus*, *M. defluvii*, *M. thermoflexus*, *M. thermophilus* and *M. marburgensis*. The strains *M. tenebrarum* and *M. crinale* cluster in a separate clade of the phylogenetic tree and have a larger distance to the *M. wolfeii* SIV6 strain. The chosen outgroup is *Methanobacterium formicicum* since the genus *Methanobacterium* is closely related to the genus *Methanothermobacter*. To date, three *Methanothermobacter* strains, namely *Methanothermobacter* sp. CaT2, *M. thermautotrophicus* ΔH and *M. marburgensis* str. Marburg, were completely sequenced. ANI (average nucleotide identity) calculations for these three *Archaea* revealed values between 84.72% and 95.75% (Figure 3). The comparison of *M. wolfeii* SIV6 with the reference strains showed ANI values between 83.07% and 83.89%. Due to these results, strain SIV6 is the most distant relative on genus level. ANI values of above 95% indicate organisms belonging to the same species [42], but there is no ANI cutoff for genus level.

### 3.3. Genome Features of M. wolfeii SIV6 in Combination with In Situ Genome-Centered Metatranscriptomics

In the following subchapters genome features of *M. wolfeii* SIV6, as deduced from genome analysis and comparative analysis, are analyzed regarding their potential beneficial functions for the strain. To get insights into the transcriptional activity of these genes in situ, genome-centered metatranscriptome mappings are included. This approach resulted in mapping of about 2 million quality-controlled metatranscriptome reads of the corresponding thermophilic biogas plant on the *M. wolfeii* genome and the calculation of transcripts per million (TPM) values for 1784 of the 1786 annotated genes. The TPM values of these genes range from 0.5 up to 74,807.6, with a mean TPM value of 561 and a median of 136.

#### 3.3.1. Functional Genome Annotation in Combination with Transcriptional Activity of Genes

**Figure 4:**
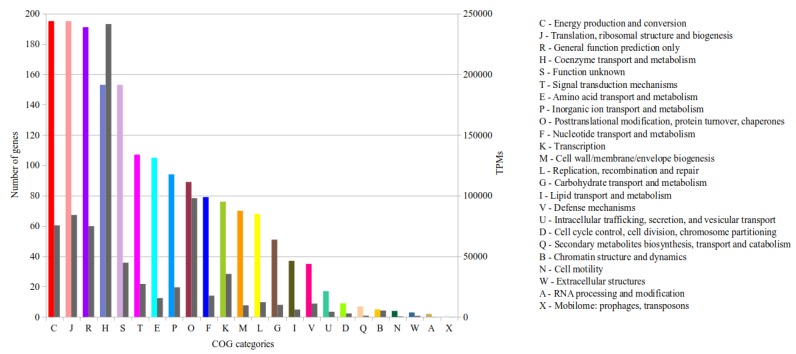
Functional classification of the *M. wolfeii* SIV6 genes and their corresponding TPM according to COG (Clusters of Orthologous Groups). Shown are the COG categories (X-axis), the number of genes belonging to each category (left Y-axis) colored according to the COG categories and the TPM belonging to each category (right Y-axis) colored in grey.

In total, 1784 genes were functionally annotated by means of WebMGA [29]. 1498 of these genes were assigned to 24 different Clusters of Orthologous Group (COG) categories (Figure 4) and 286 genes remain unassigned. To get insights into the potential metabolic activity under the conditions prevailing in the analyzed biogas fermenter, the TPM of each COG category were summed up (Figure 4). The COG categories C (‘Energy production and conversion’), J (‘Translation, ribosomal structure and biogenesis’), R (‘General function prediction only’) and H (‘Coenzyme transport and metabolism’) have the most assigned genes (Figure 4) with 11.2%, 11.2%, 10.9% and 8.8% of all COG-assigned genes. Considering the transcriptional activity, the most TPM (241,360) belong to the category H. In this group, the highest transcribed genes are *mcr*, *mtr* and *mer*, which belong to the hydrogenotrophic methanogenesis pathway (see Section 3.3.4). In general, little is known about the in situ transcriptional activity of single archaeal strains in biogas fermenters. However, it was shown for microbial communities in biogas fermenters, that archaeal genes involved in energy metabolism and methanogenesis were among the highest transcribed genes [43] and corresponding gene products are highly expressed [44,45,46].

The second most TPM (97,901) belong to category O (’post-translational modification, protein turnover, and chaperones’). The two highest transcribed genes within this group have no predicted functions, but account for 65,273 and 7385 TPM of this category. The third highest transcribed gene (2604 TPM) encodes a proteasome-activating nucleotidase (PAN) representing a protein-unfolding molecular chaperone [47], which matches perfectly the COG category O.

#### 3.3.2. Strain Specific Genome Features as Deduced from Singleton Analyses

The core genome of *M. wolfeii* SIV6 and the reference strains *Methanothermobacter* sp. CaT2, *M. thermautotrophicus* ΔH and *M. marburgensis* str. Marburg, which is based on calculations within the comparative genomics tool EDGAR [31], consists of 1494 orthologous coding sequences (Figure S1). Thus, 90% of all coding sequences of *M. wolfeii* SIV6 belong to the core genome. Seventy-four genes were identified as singletons of strain SIV6, which are genes without any hit against any of the reference genomes [31]. Fifty-eight of these genes encode hypothetical proteins and the remaining 16 singletons encode proteins with predicted functions (Table 3).

Five of the identified singletons (singleton number 1, 2, 4, 5, 8) encode glycosyltransferases (GTs) (EC 2.4.-.-) representing enzymes involved in protein glycosylation, catalyzing the transfer of sugar moieties from activated donor molecules to specific acceptor molecules. Thus, they are involved in forming of glycosidic bonds during the biosynthesis of disaccharides, oligosaccharides and polysaccharides. These saccharides are then covalently linked to either asparagine (N-glycosylation) or serine and threonine residues (O-glycosylation) [48]. In contrast to *Bacteria*, where N-glycosylation is a rare event, it is a common post-translational modification in *Archaea* [49]. Almost all sequenced archaeal genomes harbour the key enzyme for N-glycosylation, the oligosaccharyl-transferase AglB [48]. This enzyme was also identified in *M. wolfeii* SIV6 (MSIV6_0432) and the reference strain *M. thermautotrophicus* ΔH (MTH_RS09100; NCBI-ID: 1071). One of the identified singleton glycosyltransferases belongs to the glycosyltransferase family 2 (GT2) and the remaining four singletons to the glycosyltransferase family 4 (GT4) (Table 4). The families GT2 and GT4 are large GT families with a broad range of different catalytic activities, including many steps in N-glycosylation pathways [50]. The majority of archaeal cell walls consists of a proteinaceous surface layer (S-layer) as only component. The S-layer proteins and other surface-exposed proteins (e.g., archaellins, sugar-binding proteins) are post-translationally modified by glycosylation [48,51]. This modification influences the maintenance of cell integrity and cell stability, as well as folding, stability and assembly of the surface-exposed proteins [52,53]. Among the reference strains, *M. wolfeii* SIV6 possesses the most glycosyltransferases (Table 4), of which five represent singletons. Additionally, all singleton glycosyltransferases showed transcriptional activity and in particular the hexosyltransferase (singleton number 4) has with 520 the highest TPM value among all singletons of *M. wolfeii* (Table 3). Thus, *M. wolfeii* SIV6 and especially its cell wall could be better adapted to harsh environments like thermophilic biogas fermenters compared to the reference strains.

**Figure 5:**
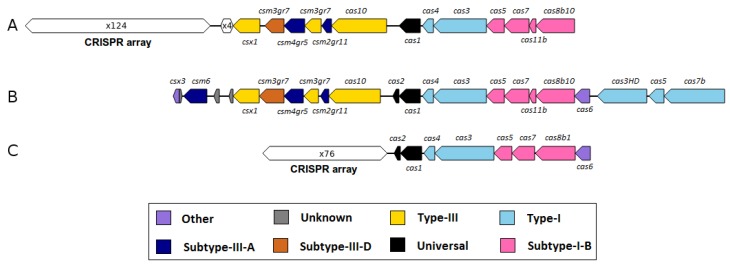
Comparison of a partly homologous CRISPR/*cas* system within the *M. thermautotrophicus* ΔH (**A**), *M. wolfeii* SIV6 (**B**) and *Methanothermobacter* sp. CaT2 (**C**) genomes. Shown are the *cas* gene types in different colors and the CRISPR arrays with the number of repeats. CRISPR arrays were identified with CRISPRone [56].

Furthermore, the singleton analysis revealed four genes (singleton number 13, 14, 15, 16: CRISPR-associated protein Csx1, CRISPR-associated nuclease/helicase Cas3, CRISPR-associated protein Cas5, CRISPR-associated protein Cas7, respectively) that are associated with type I and III CRISPR/*cas* systems (Clustered Regularly Interspaced Short Palindromic Repeats/CRISPR-associated system). CRISPR/*cas* represent defense systems of many *Bacteria* and most *Archaea* against viral infections and the exposure of invading nucleic acids. Therefore, the hypervariable CRISPR arrays harbor genetic signatures from invasive elements which lead to inheritable DNA-encoded immunity [54]. A detailed analysis of these CRISPR/*cas* associated genes within the *M. wolfeii* genome revealed three CRISPR arrays with 33, 74 and 4 repeats and two *cas* gene clusters. The reference strains also harbor CRISPR/*cas* associated genes. Strain *Methanothermobacter* sp. CaT2 has three CRISPR arrays and four *cas* gene clusters, one of which is directly associated with a CRISPR array. *M. marburgensis* str. Marburg has two CRISPR arrays and one *cas* gene cluster. *M. thermautotrophicus* ΔH has three CRISPR arrays and three *cas* gene clusters, one of which is directly associated with a CRISPR array. The four CRISPR/*cas* associated singletons of strain SIV6 are located up- and downstream of a *cas* gene locus which is also present in *M. thermautotrophicus*ΔH and *Methanothermobacter* sp. CaT2 (Figure 5). Zhang and Ye (2017) [55] defined a *cas* locus as containing at least three *cas* genes, with at least one of the universal *cas* genes (*cas*1, *cas*2) or one of the main components of interference modules (*cas*7, *cas*5, *cas*8, *cas*10, *cas*1, *cas*9, *cas*1). Usually, *cas* gene loci are located in direct vicinity to CRISPR arrays. If no CRISPR arrays are located in close proximity of a *cas* locus, the module is called an isolated *cas* locus. These isolated *cas* loci can either be non-functional or function together with a distant CRISPR array in the same genome [55]. The *cas* gene clusters of strain SIV6 harbor *cas*1, *cas*2, *cas*5, *cas*7 and the *cas*10 gene, specifying these *cas* loci as type I and III (Figure 5). These loci do not cluster in the flanking regions of the CRISPR arrays, but metatranscriptome analysis of these genes showed that about 0.42% of all TPM map onto the *cas* genes of *M. wolfeii* SIV6 indicating activity of theses CRISPR/*cas* systems. Due to these results, *M. wolfeii* SIV6 seems to have a functional defense system against viral infections and invading nucleic acids. Presence of CRISPR/*cas* systems in the *M. wolfeii* genome and their transcriptional activity is in accordance with the fact that no phage-associated genes or regions were identified in the genome with PHASTER [41]. In contrast, the *M. marburgensis* str. Marburg genome harbours one incomplete prophage region consisting of 8 phage associated genes and the *M. thermautotrophicus*ΔH genome harbours one incomplete prophage region consisting of 20 phage associated genes. In the *Methanothermobacter* sp. CaT2 genome, no phage associated genes or regions could be identified.

Additionally, the singleton analysis revealed a gene encoding a Phycobilisome (PBS) lyase HEAT domain protein (singleton number 11). This gene was already identified in the chemotaxis gene regions of all motile *Haloarchaea* species but not in other archaeal species so far [57]. In these *Haloarchaea* species, deletion of this gene resulted in mutants, which were only able to swim forward and were unable to respond to signals [57]. In contrast, in the genome of *M. wolfeii* SIV6, the PBS lyase HEAT domain protein is not linked with chemotaxis or flagellar motility, since *M. wolfeii* does not harbor any chemotaxis genes (*che*) or flagellar accessory genes (*fla*) like motile archaeal species [58]. Moreover, this singleton is not transcribed as deduced from metatranscriptome analyses and the gene is located on a genomic island region within the *M. wolfeii* genome (Table 3). Therefore, it can be assumed that this gene was integrated via horizontal gene transfer and that it does not have a functionality within the *M. wolfeii* genome. The remaining six singletons (singleton number 3, 6, 7, 9, 10, 12) of *M. wolfeii* SIV6 showed rather general metabolic functions (e.g., DNA synthesis, transport of protons/cations/ions) and rather low TPM values between 2 and 79, thus their predicted functions are summarized in Table 3.

#### 3.3.3. Genomic Islands and Restriction-Modification Systems as Additional Genome Features of *M. wolfeii* SIV6

Genomic islands often comprise clusters of genes featuring corporate functions and/or a shared evolutionary background [59]. These gene clusters are suggested to be integrated into the genome via horizontal gene transfer and can have different and often adaptive functions like defense mechanisms, metabolism related functions, resistance mechanisms and others [27,59,60,61]. In the genome of *M. wolfeii* SIV6, three genomic island regions were identified (GI 1–3, Figure 1). Analyses based on REBASE (The Restriction Enzyme Database [62]) showed that the first genomic island region (GI 1) comprising three genes, encodes a potential restriction-modification system (RM system). RM systems are defense systems of prokaryotes against foreign DNA and commonly consist of a restriction endonuclease (R), which cleaves DNA and a methyltransferase (M), which methylates the own DNA to protect it against cleavage by the restriction endonuclease [63]. The identified potential Type I RM system of the genomic island consists of three subunits: R subunit, M subunit and a site-specific (S) subunit. These subunits build a complex that is able to cleave and methylate DNA [64]. Thus, this genomic island represents a potential defense island of *M. wolfeii* SIV6. In addition, two more potential RM systems were identified in the *M. wolfeii* SIV6 genome with REBASE. The second one (861,168–867,135) is a potential Type II RM system consisting of an R and M enzyme that operate independently of each other for cleavage and methylation of DNA. The third one (1,196,654–1,197,175) is a potential Type IV RM system only featuring an R component, which is able to recognize and hydrolyze modified DNA with a low specificity. This allows protection against a broad range of foreign DNA with different methylation patterns [65]. The second genomic island region (GI 2) harbors eleven genes encoding hypothetical proteins and three genes with predicted functions (PBS lyase HEAT domain protein, UV radiation resistance protein and autophagy-related subunit 14, Integrase family protein). The PBS lyase HEAT domain protein has a predicted function in chemotaxis and motility of archaeal strains [57] but, as already discussed in Section 3.3.2, seems to be non-functional in the *M. wolfeii* genome. The genes encoding the potential UV radiation resistance protein and autophagy-related subunit 14 and the potential integrase family protein also seem to be non-functional in the *M. wolfeii* genome since they were almost not transcribed under in situ fermenter conditions. Thus, for this genomic island no potential function can be predicted. The third genomic island region (GI 3) comprises three CRISPR/*cas*-associated genes (*cas*3, *cas*5, *cas*7). These genes are located upstream and downstream of an existing CRISPR/*cas* gene cluster as described in Section 3.3.2 (Figure 5). Due to the adaptive immunity function of CRISPR/*cas* systems, this genomic island can be predicted as potential defense island. In conclusion, the genome of *M. wolfeii* SIV6 harbours three genomic islands of which two were predicted as potential defense islands against foreign DNA invasion such as phage infections. Phages were shown to occur in biogas fermenters and have a major role in shaping of the microbial community [56,66]. The described defense mechanisms of *M. wolfeii* SIV6 could explain the abundance and competitiveness of this strain in the microbial community of the analyzed biogas fermenter.

#### 3.3.4. Reconstruction and Transcriptional Activity of the Hydrogenotrophic Methanogenesis Pathway of *M. wolfeii* SIV6

**Figure 6:**
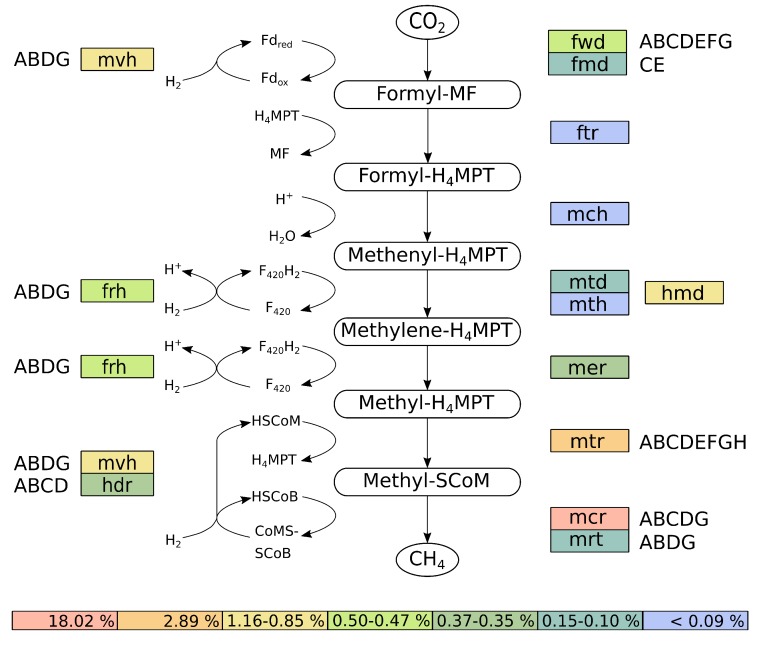
Reconstructed hydrogenotrophic methanogenesis pathway of *M. wolfeii* SIV6. Colors indicate the percentage of TPM (transcripts per million) values belonging to these genes or operons. Subunits of enzymes are indicated by the capital letters next to the corresponding gene designation. Abbreviations are further explained in the text.

Further analyses of the transcriptional activity of *M. wolfeii* SIV6 showed, that the highest transcribed genes of *M. wolfeii* belong to the hydrogenotrophic methanogenesis pathway, with *mcr*D as the highest transcribed gene (74,808 TPM, Table 5). This gene belongs to the *mcr* operon, which catalyzes the final step in the methanogenesis pathway. Thus, the hydrogenotrophic methanogenesis pathway was reconstructed, considering the TPM values of the involved genes (Figure 6).

The reconstruction of the hydrogenotrophic methanogenesis pathway was performed with KAAS [38]. Therefore, the genes were mapped onto the corresponding methanogenesis pathway originating from the KEGG database and the corresponding TPM values of the assigned genes were summed up (Figure 6). *M. wolfeii* SIV6 uses the substrate CO${}_{2}$ and the electron donor H${}_{2}$ for the hydrogenotrophic methanogenesis pathway. Additionally *M. wolfeii* SIV6 is able to use formate as substrate for methanogenesis, which is oxidised by formate dehydrogenases (FdhA-D, 729 TPM) to CO${}_{2}$ (Wasserfallen et al., 2000; Wood et al., 2003). The hydrogenotrophic methanogenesis pathway consists of seven steps (Figure 6) [67]. The first step is the reduction of CO${}_{2}$ to formyl-methanofuran (formyl-MF) with ferredoxin (Fd${}_{red}$) as electron donor. This step is catalyzed by two enzymes: a molybdenum- and tungsten-dependent formylmethanofuran dehydrogenase (FmdCE, 1084 TPM; FwdA-G, 5210 TPM). Afterwards the formyl group is transferred to tetrahydromethanopterin (HMPT) yielding formyl-H${}_{4}$MPT, catalyzed by a formyltransferase (Ftr, 275 TPM). The following dehydration of formyl-H${}_{4}$MPT is catalyzed by methenyl-H${}_{4}$MPT cyclohydrolase (Mch, 143 TPM). The fourth step is the reduction of methenyl-H${}_{4}$MPT to methylene-H${}_{4}$MPT. This step is catalyzed by the iso-functional enzymes methylene-H${}_{4}$MPT dehydrogenase (Hmd, 11,581 TPM) which utilizes H${}_{2}$ as electron donor (Goldman et al., 2009) or methylene-H${}_{4}$MPT dehydrogenase (Mtd, 1523 TPM; Mth, 120 TPM), which uses F${}_{420}$H${}_{2}$ as electron donor (Figure 6). The following reduction step is catalyzed by methylene-H${}_{4}$MPT reductase (Mer, 3477 TPM), where F${}_{420}$H${}_{2}$ is used as electron donor, yielding methyl-H${}_{4}$MPT. During the sixth step, the methyl group of methyl-H${}_{4}$MPT is transferred to coenzyme M (HSCoM) by methyl-H${}_{4}$MPT methyltransferase (MtrA-H, 28,896 TPM). Methyl-SCoM is finally reduced to methane with coenzyme B (HSCoB) as electron donor. This final and rate-limiting step of the methanogenesis pathway [68] is catalyzed by the iso-functional enzymes methyl-coenzyme M reductase I or II (McrA-DG, 180,239 TPM; MrtABDG, 1019 TPM). With *mcr* as the highest transcribed operon of the *M. wolfeii* genome. The involved coenzymes are recycled concomitantly to the methanogenesis pathway. F${}_{420}$-reducing hydrogenase (FrhABDG, 4917 TPM) catalyzes the reduction of coenzyme F${}_{420}$ with H${}_{2}$. The coenzyme M and coenzyme B heterodisulfide (CoMSSCoB) is recycled by a complex of methyl-viologen-reducing hydrogenase and heterodisulfide reductase (MvhABDG, 8469 TPM; HdrA-D, 3699 TPM). This exergonic reaction is coupled with the endergonic reduction of oxidized ferredoxin (Fd${}_{ox}$) via flavin-based electron bifurcation [69].

Summation of all TPM values belonging to genes of the hydrogenotrophic methanogenesis pathway (Figure 6) showed that 25.18% of all TPM belong to these genes, with 18.02% of the TPM belonging exclusively to the *mcr* operon, followed by the *mtr* operon with 2.89% and *hmd* with 1.16%. This high transcription of the methanogenesis genes indicates a high metabolic activity of *M. wolfeii* SIV6 within the thermophilic biogas fermenter. These findings correlate with results obtained by other studies where metatranscriptomes and metaproteomes of biogas fermenters were analyzed and methanogenesis genes belong to the highest transcribed genes and methyl-coenzyme M reductase subunits were among the most abundant proteins of methanogenic *Archaea* [43,46,70]. The hydrogenotrophic methanogenesis pathway is described to be regulated by H${}_{2}$ limitation [71]. It was shown that genes encoding enzymes catalyzing oxidation and reduction of coenzyme F${}_{420}$ (*frh*, *mtd*, *mer*) were upregulated under H${}_{2}$ limitation and it was suggested that this upregulation maintains the electron flow for the methanogenesis pathway [72]. Additionally, it was shown for the iso-functional enzymes Mtd and Hmd, which catalyze the fourth step of the hydrogenotrophic methanogenesis, that Mtd increases and Hmd decreases under H${}_{2}$ limiting conditions and that Hmd is preferred when H${}_{2}$ is in excess [73]. In the hydrogenotrophic methanogenesis pathway of *M. wolfeii*, the TPM value of Hmd is 7-fold higher in contrast to the TPM value of Mtd/Mth (Figure 6). Hence, it is suggested that no H${}_{2}$ limitation was prevalent within the analyzed thermophilic biogas fermenter and/or *M. wolfeii* is syntrophically connected with H${}_{2}$ supplying bacteria. Syntrophic interactions and especially the syntrophic hydrogen transfer between *Bacteria* and hydrogenotrophic *Archaea* are often described in the context of the biogas process [74,75,76]. Syntrophic growth of *M. thermautotrophicus* with *Syntrophothermus lipocaldicus* revealed, that the methyl-coenzyme M reductase I (Mcr) was preferred instead of methyl-coenzyme M reductase II (Mrt), in contrast to expression of both enzymes in pure culture of *M. thermautotrophicus* [77]. In the hydrogenotrophic methanogenesis pathway of *M. wolfeii*, the TPM value of *mcr* is 176-fold higher than the TPM value of *mrt* (Figure 6). Hence, as already mentioned above, it is suggested, that *M. wolfeii* is syntrophically connected to H${}_{2}$ supplying bacteria within the thermophilic biogas fermenter.

### 3.4. Fragment Recruitment of Metagenomic Reads from the Corresponding Thermophilic Biogas Fermenter on the M. wolfeii SIV6 Genome

To gain insights into the abundance of *M. wolfeii* strain SIV6 in the corresponding thermophilic biogas, a fragment recruitment was performed (Figure S2). For this purpose, about 228 million quality-controlled metagenomic reads obtained for the microbiome of the thermophilic biogas fermenter were mapped to the *M. wolfeii* SIV6 genome sequence. The fragment recruitment yielded 1,640,767 reads with a sequence identity above 75% and 1,521,029 reads with a sequence identity above 97% in mappings to the *M. wolfeii* genome, representing 0.7% and 0.6% of all metagenomic reads, respectively. About half of these (889,284 reads) featured a perfect match (100% sequence identity) with the *M. wolfeii* genome. Overall, the fragment recruitment analysis revealed an abundance of 1.2% (all reads with sequence identity above 55%) of *M. wolfeii* SIV6 within the thermophilic biogas fermenter. Additionally, the fragment recruitment analysis revealed that the *M. wolfeii* SIV6 genome is almost completely covered by mapped metagenomic reads. However, one gap of about 20 kb was noticed in the genome coverage at position 1196 to 1214 kb. This gap corresponds a region of 19 genes representing the regions GI 1 and 2 with one additional gene (*mrr*, MSIV6_1287) encoding a restriction endonuclease of a restriction-modification system IV, upstream of GI 1 and one gene encoding a hypothetical protein between GI 1 and GI 2. Thus, it could be possible, that slightly different *M. wolfeii* strains were present within the thermophilic biogas fermenter which could be differentiated by the presence or absence of the GI 1 and 2 regions. This hypothesis can be confirmed by further isolation experiments.

## 4. Conclusions

Compilation of the *M. wolfeii* SIV6 genome sequence and comparative genome analyses provided the basis for genome-centered analyses of the strain’s transcriptional activity under in situ conditions in a production-scale biogas fermenter by employing a metatranscriptomics approach. Accordingly, the *M. wolfeii* SIV6 performance was studied in a ‘real-life’ environment. Obtained results complement and substantiate current knowledge on the functioning of a *Methanothermobacter* species within the complex microbial community of a thermophilic biogas process. Our analysis revealed that *M. wolfeii* SIV6 possesses genetic features that may mediate a better competitiveness of this strain in environments like thermophilic biogas fermenters. On the one hand, *M. wolfeii* harbours 29 genes that encode glycosyltransferases of which five are singleton genes. Glycosylation is an important protection of proteins against degradation and conformational changes. Furthermore, four CRISPR/*cas* associated singletons were identified within the *M. wolfeii* genome. CRISPR/*cas* systems are a common defense mechanism of *Bacteria* and *Archaea* providing immunity against phages and invading nucleic acids. Additionally, the *M. wolfeii* genome analysis revealed all necessary genes for the hydrogenotrophic methanogenesis pathway. Genome-centered metatranscriptomics showed that about one fourth of all TPM mapped on the genome correspond to genes featuring predicted functions in the hydrogenotrophic methanogenesis pathway. About 18% of these TPM values exclusively belong to the *mcr* operon which encodes the different subunits of methyl-coenzyme M reductase. This enzyme catalyzes the final and rate-limiting step during methanogenesis. Detailed genome analyses of single microorganisms, especially of archaeal strains, and their in situ transcriptional activity contributes to better understanding of their requirements and ecological roles within the biogas process. In the future, a microbial-based process management [78] and thus an optimization of the performance and stability of the biogas process is intended. In recent research, selected methanogenic *Archaea* were used in electro-methanogenesis experiments pursuing the aim to reduce carbon dioxide to methane by electric current at a biocathode [79,80,81]. This ‘power-to-gas’ strategy is of great importance regarding conversion of electrical power into the storable energy carrier methane. *Methanothermobacter* spp. were previously identified to dominate biocathode microbiomes of thermophilic bioelectrochemical systems (BES) [81,82,83]. Therefore, we suggest testing and application of the *Methanothermobacter wolfeii* SIV6 isolate in electro-methanogenesis experiments since it is well adapted to thermophilic conditions.

## Figures and Tables

**Table 1:** Overview of all known *Methanothermobacter* species according to the `list of prokaryotic names with standing in nomenclature’ ${}^{1}$, their origin and reference.

Species	Strain	Origin	Reference
*M. crinale*	Tm2${}^{T}$	Oil field	Cheng et al. (2011) [13]
*M. defluvii*	ADZ${}^{T}$	Digester sludge	Kotelnikova et al. (1993) [14]
*M. marburgensis*	Marburg${}^{T}$	Sewage sludge	Liesegang et al. (2010) [15]
*M. thermautotrophicus*	ΔH${}^{T}$	Sewage sludge	Zeikus and Wolfe (1972) [16]
*M. thermoflexus*	IDZ${}^{T}$	Digester sludge	Kotelnikova et al. (1993) [14]
*M. thermophilus*	M${}^{T}$	Thermophilic methane tank	Laurinavichus et al. (1988) [17]
*M. tenebrarum*	RMAS${}^{T}$	Gas field	Nakamura et al. (2013) [18]
*M. wolfeii*	JCM 14652${}^{T}$	Sewage sludge	Winter et al. (1984) [19]

${}^{1}$ Parte, 2018.

**Table 2:** General genome features of *M. wolfeii* SIV6.

General Features	*M. wolfeii* SIV6
Size (bp)	1,686,891
GC content (%)	48.89
Total genes	1786
Protein coding genes	1659
Genes assigned to COG ${}^{1}$ categories	1498
*rrn* operons	2
tRNA genes	36
Genomic islands	3

${}^{1}$ COG—Cluster of Orthologous Group.

**Table 3:** The *M. wolfeii* SIV6 singletons encoding proteins with a predicted function and their transcripts per million (TPM) values.

Singleton Number	Locus	Gene Annotation ${}^{1}$	Predicted Function	GI ${}^{2}$	TPM ${}^{3}$
1	MWSIV6_ 0587	Glycosyltransferase	protein glycosylation	-	22
2	MWSIV6_ 0588	Glycosyltransferase	protein glycosylation	-	20
3	MWSIV6_ 0666	4Fe-4S ferredoxin	mediating the transfer of electrons	-	10
			in different metabolic reactions		
4	MWSIV6_ 0722	Hexosyltransferase	protein glycosylation	-	520
5	MWSIV6_ 0726	Glycosyltransferase	protein glycosylation	-	202
6	MWSIV6_ 0728	Thymidylate kinase	DNA synthesis	-	29
7	MWSIV6_ 0729	Alkaline Phosphatase	post-translational modification	-	47
8	MWSIV6_ 0732	Uncharacterized	protein glycosylation	-	52
		Glycosyltransferase			
9	MWSIV6_ 0863	ATPase	drives the transport of protons or	-	79
			other cations across the cell membrane		
10	MWSIV6_ 0990	Cl-channel voltage-	transfers chloride ions	-	26
		gated family protein	across the membrane		
11	MWSIV6_ 1297	PBS ${}^{4}$ lyase HEAT	archaeal chemotaxis	GI 2	0
		domain protein			
12	MWSIV6_ 1305	Integrase family protein	DNA breaking and rejoining	GI 2	2
13	MWSIV6_ 1466	CRISPR-associated	defense system	-	33
		protein Csx1			
14	MWSIV6_ 1483	CRISPR-associated	defense system	GI 3	143
		nuclease/helicase Cas3			
15	MWSIV6_ 1484	CRISPR-associated	defense system	GI 3	126
		protein Cas5			
16	MWSIV6_ 1485	CRISPR-associated	defense system	GI 3	300
		protein Cas7			

${}^{1}$ Hypothetical genes without functional annotation are not shown; ${}^{2}$ Genomc Island; ${}^{3}$ Transcripts per million (TPM) values were generated by mapping of the metatranscriptome reads of the corresponding biogas fermenter onto the *M. wolfeii* SIV6 genome; ${}^{4}$ Phycobilisomes

**Table 4:** Comparison of carbohydrate active enzymes of *M. wolfeii* SIV6 and the reference strains *Methanothermobacter* sp. CaT2, *M. thermautotrophicus* ΔH and *M. marburgensis* str. Marburg.

Glycosyltransferase Family	1	2	4	20	66	81	NC ${}^{1}$	Total
*M. wolfeii* SIV6	1	7	13	1	5	1	1	29
*Methanothermobacter* sp. CaT2	1	9	8	1	4	1	1	25
*M. thermautotrophicus*ΔH	1	9	9	1	4	1	0	25
*M. marburgensis* str. Marburg	1	7	8	1	3	1	1	22

${}^{1}$ Non classified glycosyltransferases.

**Table 5:** The ten highest transcribed genes of *M. wolfeii* SIV6, their predicted product and transcripts per million (TPM) values.

Gene ${}^{1}$	Predicted product	TPM ${}^{2}$
*mcr*D	Methyl-coenzyme M reductase I operon protein D	74,808
*mcr*C	Methyl-coenzyme M reductase I operon protein C	40,551
*mcr*B	Methyl-coenzyme M reductase I subunit beta	27,412
*mcr*G	Methyl-coenzyme M reductase I subunit gamma	21,785
*mcr*A	Methyl-coenzyme M reductase I subunit alpha	15,684
*hmd*	5,10-methylenetetrahydromethanopterin reductase	11,581
*sod*	DNA-directed RNA polymerase subunit HD	8847
*mtr*A1	F420-non-reducing hydrogenase	5997
*mtr*F	Proteasome-activating nucleotidase	5729
*mtr*G	50S ribosomal protein L29P	4138

${}^{1}$ Hypothetical genes without functional annotation are not shown. ${}^{2}$ Transcripts per million (TPM) values were generated by mapping of the metatranscriptome reads of the corresponding biogas fermenter onto the *M. wolfeii* SIV6 genome.

## Supplementary Materials

The following are available online at https://www.mdpi.com/2076-2607/8/1/13/s1, Table S1: The 50 highest transcribed genes of *M. wolfeii* SIV6, their locus, EC-number, predicted product, transcripts per million (TPM) and COG category. Figure S1: Venn diagram depicting the core genome of 1494 orthologous coding sequences (CDS) of *M. wolfeii* SIV6 and the reference strains *Methanothermobacter* sp. CaT2, *M. thermautotrophicus* ΔH and *M. marburgensis* str. Marburg based on calculations within the EDGAR platform. Figure S2: Fragment recruitment of metagenome sequences of the thermophilic biogas fermenter on the genome of *M. wolfeii* SIV6. Shown are the mapped metagenome reads based on their percent identity (y-axis) and the *M. wolfeii* genome position (x-axis).

## References

[1] Zinder S.H. (1993). Physiological ecology of methanogens. Methanogenesis.

[2] Conrad R. (1999). Contribution of hydrogen to methane production and control of hydrogen concentrations in methanogenic soils and sediments. FEMS Microbiol. Ecol..

[3] Wirth R., Kovács E., Maróti G., Bagi Z., Rákhely G., Kovács K.L. (2012). Characterization of a biogas-producing microbial community by short-read next generation DNA sequencing. Biotechnol. Biofuels.

[4] Stolze Y., Bremges A., Rumming M., Henke C., Maus I., Pühler A., Sczyrba A., Schlüter A. (2016). Identification and genome reconstruction of abundant distinct taxa in microbiomes from one thermophilic and three mesophilic production-scale biogas plants. Biotechnol. Biofuels.

[5] Evans P.N., Boyd J.A., Leu A.O., Woodcroft B.J., Parks D.H., Hugenholtz P., Tyson G.W. (2019). An evolving view of methane metabolism in the Archaea. Nat. Rev. Microbiol..

[6] Ferrer P., Cambra-López M., Cerisuelo A., Peñaranda D.S., Moset V. (2014). The use of agricultural substrates to improve methane yield in anaerobic co-digestion with pig slurry: Effect of substrate type and inclusion level. Waste Manag..

[7] Ziganshin A.M., Liebetrau J., Pröter J., Kleinsteuber S. (2013). Microbial community structure and dynamics during anaerobic digestion of various agricultural waste materials. Appl. Microbiol. Biotechnol..

[8] Maus I., Koeck D.E., Cibis K.G., Hahnke S., Kim Y.S., Langer T., Kreubel J., Erhard M., Bremges A., Off S. (2016). Unraveling the microbiome of a thermophilic biogas plant by metagenome and metatranscriptome analysis complemented by characterization of bacterial and archaeal isolates. Biotechnol. Biofuels.

[9] Yu D., Kurola J., Lähde K., Kymäläinen M., Sinkkonen A., Romantschuk M. (2014). Biogas production and methanogenic archaeal community in mesophilic and thermophilic anaerobic co-digestion processes. J. Environ. Manag..

[10] Hassa J., Maus I., Off S., Pühler A., Scherer P., Klocke M., Schlüter A. (2018). Metagenome, metatranscriptome, and metaproteome approaches unraveled compositions and functional relationships of microbial communities residing in biogas plants. Appl. Microbiol. Biotechnol..

[11] Rabii A., Aldin S., Dahman Y., Elbeshbishy E. (2019). A review on anaerobic co-digestion with a focus on the microbial populations and the effect of multi-stage digester configuration. Energies.

[12] Parte A.C. (2018). LPSN–List of Prokaryotic names with Standing in Nomenclature (bacterio. net), 20 years on. Int. J. Syst. Evol. Microbiol..

[13] Cheng L., Dai L., Li X., Zhang H., Lu Y. (2011). Isolation and characterization of *Methanothermobacter crinale* sp. nov., a novel hydrogenotrophic methanogen from the Shengli oil field. Appl. Environ. Microbiol..

[14] Kotelnikova S., Obraztsova A.Y., Gongadze G., Laurinavichius K. (1993). *Methanobacterium thermoflexum* sp. nov. and *Methanobacterium defluvii* sp. nov., thermophilic rod-shaped methanogens isolated from anaerobic digestor sludge. Syst. Appl. Microbiol..

[15] Liesegang H., Kaster A.K., Wiezer A., Goenrich M., Wollherr A., Seedorf H., Gottschalk G., Thauer R.K. (2010). Complete genome sequence of Methanothermobacter marburgensis, a methanoarchaeon model organism. J. Bacteriol..

[16] Zeikus J., Wolfe R. (1972). Methanobacterium thermoautotrophicus should be Methanobacterium thermoautotrophicum. Int. J. Syst. Evol. Microbiol..

[17] Laurinavichus K.S., Kotelnikova S.V., Obraztsova A.Y. (1988). A new species of the thermophilic methane-forming bacterium Methanobacterium thermophilum. Mikrobiologiya.

[18] Nakamura K., Takahashi A., Mori C., Tamaki H., Mochimaru H., Nakamura K., Takamizawa K., Kamagata Y. (2013). *Methanothermobactertenebrarum* sp. nov., a hydrogenotrophic, thermophilic methanogen isolated from gas-associated formation water of a natural gas field. Int. J. Syst. Evol. Microbiol..

[19] Winter J., Lerp C., Zabel H.P., Wildenauer F., König H., Schindler F. (1984). *Methanobacterium wolfei*, sp. nov., a new tungsten-requiring, thermophilic, autotrophic methanogen. Syst. Appl. Microbiol..

[20] Kosaka T., Toh H., Toyoda A. (2013). Complete genome sequence of a thermophilic hydrogenotrophic methanogen, *Methanothermobacter* sp. strain CaT2. Genome Announc..

[21] Smith D.R., Doucette-Stamm L.A., Deloughery C., Lee H., Dubois J., Aldredge T., Bashirzadeh R., Blakely D., Cook R., Gilbert K. (1997). Complete genome sequence of Methanobacterium thermoautotrophicum deltaH: Functional analysis and comparative genomics. J. Bacteriol..

[22] Balch W., Fox G., Magrum L., Woese C., Wolfe R. (1979). Methanogens: Reevaluation of a unique biological group. Microbiol. Rev..

[23] Gordon D., Abajian C., Green P. (1998). Consed: A graphical tool for sequence finishing. Genome Res..

[24] Wibberg D., Blom J., Jaenicke S., Kollin F., Rupp O., Scharf B., Schneiker-Bekel S., Sczcepanowski R., Goesmann A., Setubal J.C. (2011). Complete genome sequencing of Agrobacterium sp. H13-3, the former Rhizobium lupini H13-3, reveals a tripartite genome consisting of a circular and a linear chromosome and an accessory plasmid but lacking a tumor-inducing Ti-plasmid. J. Biotechnol..

[25] Meyer F., Goesmann A., McHardy A.C., Bartels D., Bekel T., Clausen J., Kalinowski J., Linke B., Rupp O., Giegerich R. (2003). GenDB—An open source genome annotation system for prokaryote genomes. Nucleic Acids Res..

[26] Seemann T. (2014). Prokka: Rapid Prokaryotic Genome Annotation. Bioinformatics.

[27] Bertelli C., Laird M.R., Williams K.P., Lau B.Y., Hoad G., Winsor G.L., Brinkman F.S., Simon Fraser University Research Computing Group (2017). IslandViewer 4: Expanded prediction of genomic islands for larger-scale datasets. Nucleic Acids Res..

[28] Jia B., Raphenya A.R., Alcock B., Waglechner N., Guo P., Tsang K.K., Lago B.A., Dave B.M., Pereira S., Sharma A.N. (2016). CARD 2017: Expansion and model-centric curation of the comprehensive antibiotic resistance database. Nucleic Acids Res..

[29] Wu S., Zhu Z., Fu L., Niu B., Li W. (2011). WebMGA: A customizable web server for fast metagenomic sequence analysis. BMC Genom..

[30] Kumar S., Stecher G., Li M., Knyaz C., Tamura K. (2018). MEGA X: Molecular evolutionary genetics analysis across computing platforms. Mol. Biol. Evol..

[31] Blom J., Kreis J., Spänig S., Juhre T., Bertelli C., Ernst C., Goesmann A. (2016). EDGAR 2.0: An enhanced software platform for comparative gene content analyses. Nucleic Acids Res..

[32] Goris J., Konstantinidis K.T., Klappenbach J.A., Coenye T., Vandamme P., Tiedje J.M. (2007). DNA–DNA hybridization values and their relationship to whole-genome sequence similarities. Int. J. Syst. Evol. Microbiol..

[33] Stolze Y., Bremges A., Maus I., Pühler A., Sczyrba A., Schlüter A. (2018). Targeted in situ metatranscriptomics for selected taxa from mesophilic and thermophilic biogas plants. Microb. Biotechnol..

[34] Reddy T.B., Thomas A.D., Stamatis D., Bertsch J., Isbandi M., Jansson J., Mallajosyula J., Pagani I., Lobos E.A., Kyrpides N.C. (2014). The Genomes OnLine Database (GOLD) v. 5: A metadata management system based on a four level (meta) genome project classification. Nucleic Acids Res..

[35] Bolger A.M., Lohse M., Usadel B. (2014). Trimmomatic: A flexible trimmer for Illumina sequence data. Bioinformatics.

[36] Langmead B., Salzberg S.L. (2012). Fast gapped-read alignment with Bowtie 2. Nat. Methods.

[37] Hilker R., Stadermann K.B., Schwengers O., Anisiforov E., Jaenicke S., Weisshaar B., Zimmermann T., Goesmann A. (2016). ReadXplorer 2—Detailed read mapping analysis and visualization from one single source. Bioinformatics.

[38] Moriya Y., Itoh M., Okuda S., Yoshizawa A.C., Kanehisa M. (2007). KAAS: An automatic genome annotation and pathway reconstruction server. Nucleic Acids Res..

[39] Schlüter A., Bekel T., Diaz N.N., Dondrup M., Eichenlaub R., Gartemann K.H., Krahn I., Krause L., Krömeke H., Kruse O. (2008). The metagenome of a biogas-producing microbial community of a production-scale biogas plant fermenter analysed by the 454-pyrosequencing technology. J. Biotechnol..

[40] Eikmeyer F.G., Köfinger P., Poschenel A., Jünemann S., Zakrzewski M., Heinl S., Mayrhuber E., Grabherr R., Pühler A., Schwab H. (2013). Metagenome analyses reveal the influence of the inoculant Lactobacillus buchneri CD034 on the microbial community involved in grass ensiling. J. Biotechnol..

[41] Arndt D., Grant J.R., Marcu A., Sajed T., Pon A., Liang Y., Wishart D.S. (2016). PHASTER: A better, faster version of the PHAST phage search tool. Nucleic Acids Res..

[42] Figueras M.J., Beaz-Hidalgo R., Hossain M.J., Liles M.R. (2014). Taxonomic affiliation of new genomes should be verified using average nucleotide identity and multilocus phylogenetic analysis. Genome Announc..

[43] Zakrzewski M., Goesmann A., Jaenicke S., Jünemann S., Eikmeyer F., Szczepanowski R., Al-Soud W.A., Sørensen S., Pühler A., Schlüter A. (2012). Profiling of the metabolically active community from a production-scale biogas plant by means of high-throughput metatranscriptome sequencing. J. Biotechnol..

[44] Hanreich A., Schimpf U., Zakrzewski M., Schlüter A., Benndorf D., Heyer R., Rapp E., Pühler A., Reichl U., Klocke M. (2013). Metagenome and metaproteome analyses of microbial communities in mesophilic biogas-producing anaerobic batch fermentations indicate concerted plant carbohydrate degradation. Syst. Appl. Microbiol..

[45] Ortseifen V., Stolze Y., Maus I., Sczyrba A., Bremges A., Albaum S.P., Jaenicke S., Fracowiak J., Pühler A., Schlüter A. (2016). An integrated metagenome and-proteome analysis of the microbial community residing in a biogas production plant. J. Biotechnol..

[46] Heyer R., Benndorf D., Kohrs F., De Vrieze J., Boon N., Hoffmann M., Rapp E., Schlüter A., Sczyrba A., Reichl U. (2016). Proteotyping of biogas plant microbiomes separates biogas plants according to process temperature and reactor type. Biotechnol. Biofuels.

[47] Benaroudj N., Goldberg A.L. (2000). PAN, the proteasome-activating nucleotidase from archaebacteria, is a protein-unfolding molecular chaperone. Nat. Cell Biol..

[48] Meyer B.H., Albers S.V. (2013). Hot and sweet: Protein glycosylation in Crenarchaeota. Biochem. Soc. Trans..

[49] Calo D., Kaminski L., Eichler J. (2010). Protein glycosylation in Archaea: Sweet and extreme. Glycobiology.

[50] Breton C., Šnajdrová L., Jeanneau C., Koča J., Imberty A. (2006). Structures and mechanisms of glycosyltransferases. Glycobiology.

[51] Rodrigues-Oliveira T., Belmok A., Vasconcellos D., Schuster B., Kyaw C.M. (2017). Archaeal S-layers: Overview and current state of the art. Front. Microbiol..

[52] Lu D., Yang C., Liu Z. (2011). How hydrophobicity and the glycosylation site of glycans affect protein folding and stability: A molecular dynamics simulation. J. Phys. Chem. B.

[53] Peyfoon E., Meyer B., Hitchen P.G., Panico M., Morris H.R., Haslam S.M., Albers S.V., Dell A. (2010). The S-layer glycoprotein of the crenarchaeote Sulfolobus acidocaldarius is glycosylated at multiple sites with chitobiose-linked N-glycans. Archaea.

[54] Horvath P., Barrangou R. (2010). CRISPR/Cas, the immune system of bacteria and archaea. Science.

[55] Zhang Q., Ye Y. (2017). Not all predicted CRISPR–Cas systems are equal: Isolated cas genes and classes of CRISPR like elements. BMC Bioinform..

[56] Zhang J., Gao Q., Zhang Q., Wang T., Yue H., Wu L., Shi J., Qin Z., Zhou J., Zuo J. (2017). Bacteriophage–prokaryote dynamics and interaction within anaerobic digestion processes across time and space. Microbiome.

[57] Schlesner M., Miller A., Streif S., Staudinger W.F., Müller J., Scheffer B., Siedler F., Oesterhelt D. (2009). Identification of Archaea-specific chemotaxis proteins which interact with the flagellar apparatus. BMC Microbiol..

[58] Albers S.V., Jarrell K.F. (2018). The archaellum: An update on the unique archaeal motility structure. Trends Microbiol..

[59] Makarova K.S., Wolf Y.I., Koonin E.V. (2019). Towards functional characterization of archaeal genomic dark matter. Biochem. Soc. Trans..

[60] Makarova K.S., Wolf Y.I., Koonin E.V. (2013). Comparative genomics of defense systems in archaea and bacteria. Nucleic Acids Res..

[61] Langille M.G., Hsiao W.W., Brinkman F.S. (2010). Detecting genomic islands using bioinformatics approaches. Nat. Rev. Microbiol..

[62] Roberts R.J., Vincze T., Posfai J., Macelis D. (2014). REBASE—A database for DNA restriction and modification: Enzymes, genes and genomes. Nucleic Acids Res..

[63] Tock M.R., Dryden D.T. (2005). The biology of restriction and anti-restriction. Curr. Opin. Microbiol..

[64] Fullmer M.S., Ouellette M., Louyakis A.S., Papke R.T., Gogarten J.P. (2019). The Patchy Distribution of Restriction–Modification System Genes and the Conservation of Orphan Methyltransferases in Halobacteria. Genes.

[65] Ershova A., Rusinov I., Spirin S., Karyagina A., Alexeevski A. (2015). Role of restriction-modification systems in prokaryotic evolution and ecology. Biochemistry.

[66] Heyer R., Schallert K., Siewert C., Kohrs F., Greve J., Maus I., Klang J., Klocke M., Heiermann M., Hoffmann M. (2019). Metaproteome analysis reveals that syntrophy, competition, and phage-host interaction shape microbial communities in biogas plants. Microbiome.

[67] Reeve J.N., Nölling J., Morgan R.M., Smith D.R. (1997). Methanogenesis: Genes, genomes, and who’s on first?. J. Bacteriol..

[68] Cedervall P.E., Dey M., Pearson A.R., Ragsdale S.W., Wilmot C.M. (2010). Structural insight into methyl-coenzyme M reductase chemistry using coenzyme B analogues. Biochemistry.

[69] Buckel W., Thauer R.K. (2018). Flavin-based electron bifurcation, a new mechanism of biological energy coupling. Chem. Rev..

[70] Kougias P.G., Campanaro S., Treu L., Zhu X., Angelidaki I. (2017). A novel archaeal species belonging to Methanoculleus genus identified via de-novo assembly and metagenomic binning process in biogas reactors. Anaerobe.

[71] Cadillo-Quiroz H. (2013). Contribution of transcriptomics to systems-level understanding of methanogenic archaea. Archaea.

[72] Hendrickson E.L., Haydock A.K., Moore B.C., Whitman W.B., Leigh J.A. (2007). Functionally distinct genes regulated by hydrogen limitation and growth rate in methanogenic Archaea. Proc. Natl. Acad. Sci. USA.

[73] Xia Q., Wang T., Hendrickson E.L., Lie T.J., Hackett M., Leigh J.A. (2009). Quantitative proteomics of nutrient limitation in the hydrogenotrophic methanogen Methanococcus maripaludis. BMC Microbiol..

[74] Westerholm M., Müller B., Singh A., Karlsson Lindsjö O., Schnürer A. (2018). Detection of novel syntrophic acetate-oxidizing bacteria from biogas processes by continuous acetate enrichment approaches. Microb. Biotechnol..

[75] Theuerl S., Klang J., Prochnow A. (2019). Process disturbances in agricultural biogas production—Causes, mechanisms and effects on the biogas microbiome: A review. Energies.

[76] Schink B. (1997). Energetics of syntrophic cooperation in methanogenic degradation. Microbiol. Mol. Biol. Rev..

[77] Luo H.W., Zhang H., Suzuki T., Hattori S., Kamagata Y. (2002). Differential expression of methanogenesis genes of Methanothermobacter thermoautotrophicus (formerly Methanobacterium thermoautotrophicum) in pure culture and in cocultures with fatty acid-oxidizing syntrophs. Appl. Environ. Microbiol..

[78] Theuerl S., Herrmann C., Heiermann M., Grundmann P., Landwehr N., Kreidenweis U., Prochnow A. (2019). The future agricultural biogas plant in Germany: A vision. Energies.

[79] Zakaria B.S., Dhar B.R. (2019). Progress towards catalyzing electro-methanogenesis in anaerobic digestion process: Fundamentals, process optimization, design and scale-up considerations. Bioresource Technol..

[80] Mayer F., Enzmann F., Lopez A.M., Holtmann D. (2019). Performance of different methanogenic species for the microbial electrosynthesis of methane from carbon dioxide. Bioresource Technol..

[81] Blasco-Gómez R., Batlle-Vilanova P., Villano M., Balaguer M.D., Colprim J., Puig S. (2017). On the edge of research and technological application: A critical review of electromethanogenesis. Int. J. Mol. Sci..

[82] Fu Q., Kuramochi Y., Fukushima N., Maeda H., Sato K., Kobayashi H. (2015). Bioelectrochemical analyses of the development of a thermophilic biocathode catalyzing electromethanogenesis. Environ. Sci. Technol..

[83] Kobayashi H., Sun X., Fu Q., Maeda H., Sato K. (2017). Draft Genome Sequence of *Methanothermobacter* sp. Strain EMTCatA1, Reconstructed from the Metagenome of a Thermophilic Electromethanogenesis- Catalyzing Biocathode. Genome Announc..

