# Supplementary figures and images for: Genome Analyses and Genome-Centered Metatranscriptomics of Methanothermobacter wolfeii Strain SIV6, Isolated from a Thermophilic Production-Scale Biogas Fermenter
Source: Microorganisms. 2019 Dec 20;8(1):13. doi: 10.3390/microorganisms8010013 (PMC7022856; doi:10.3390/microorganisms8010013)

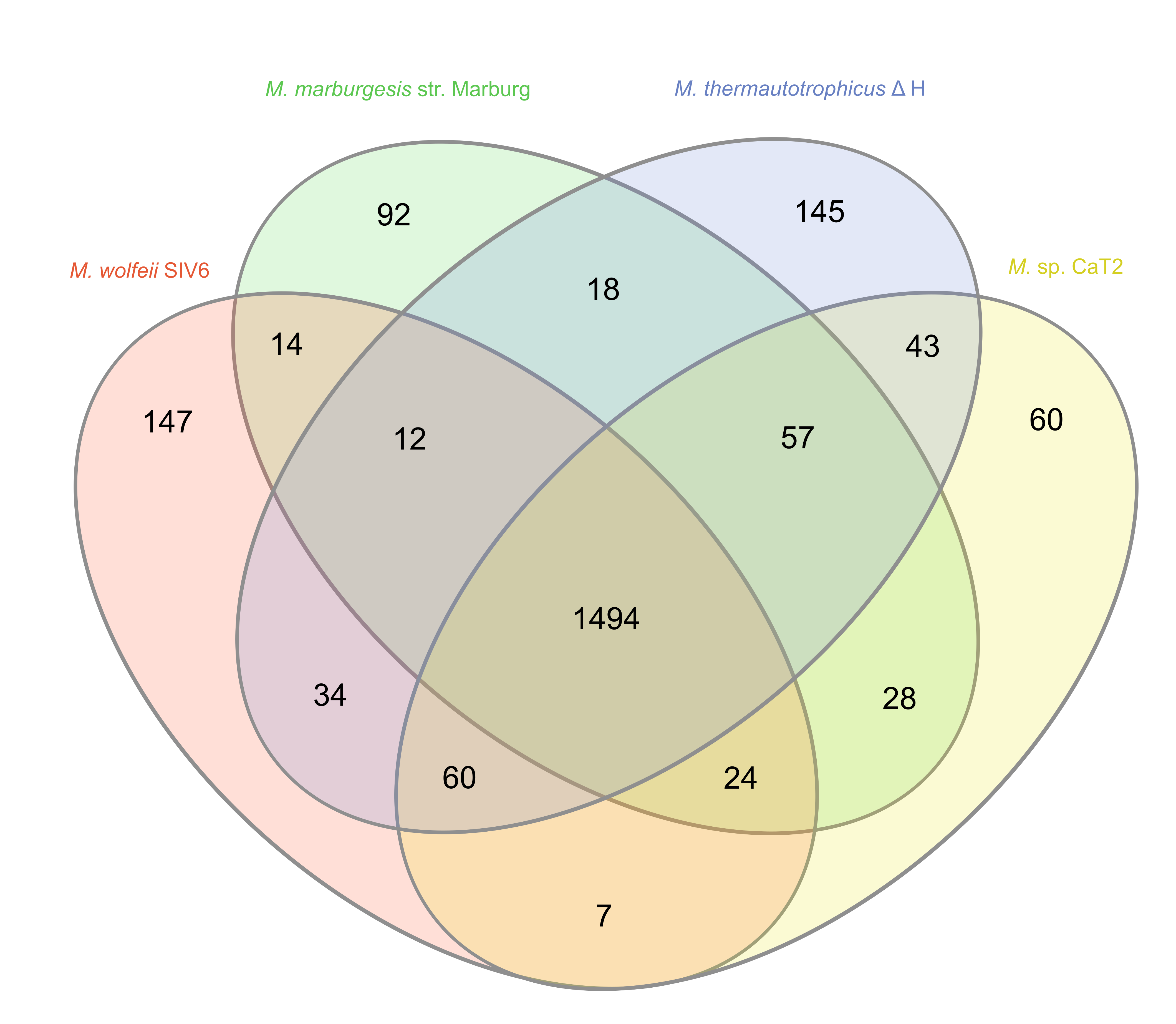

Supplement: Supplementary file 1 [file microorganisms-08-00013-s001.zip › Supplementary_Materials/Figure_S1_Venn_diagram.png]

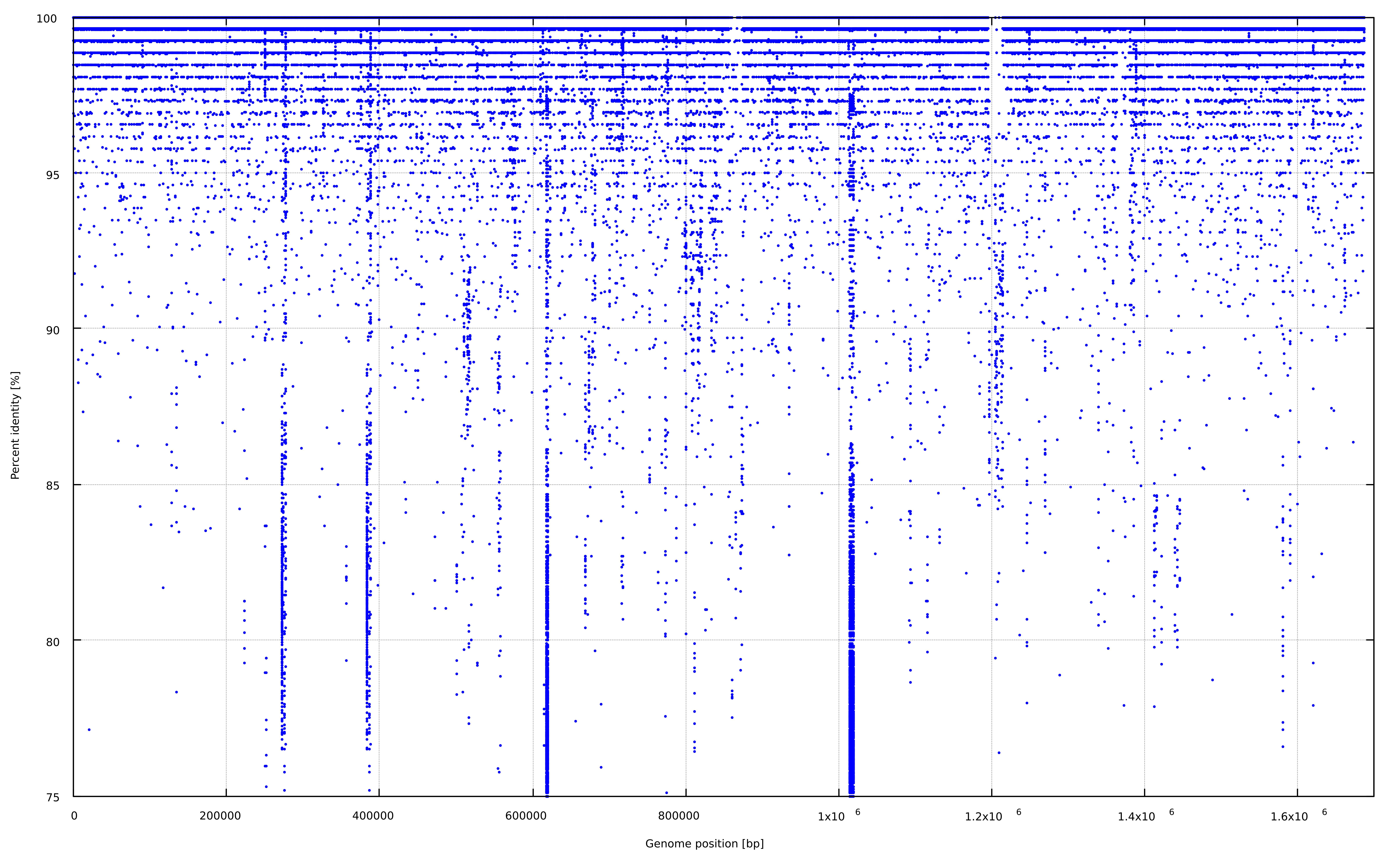

Supplement: Supplementary file 1 [file microorganisms-08-00013-s001.zip › Supplementary_Materials/Figure_S2_Fragment_recruitment.png]
